# Spontaneous suprachoroidal hemorrhage in a high myopia patient with rhegmatogenous retinal detachment: a case report and literature review

**DOI:** 10.1042/BSR20181454

**Published:** 2019-06-25

**Authors:** Fang Chai, Lu Zeng, Chunhua Li, Xiquan Zhao

**Affiliations:** Shaanxi Ophthalmic Medical Center, Xi’an No. 4 Hospital, Affiliated Guangren Hospital, School of Medicine, Xi’an Jiaotong University, Xi’an 710004, China

**Keywords:** high myopia, rhegmatogenous retinal detachment, Spontaneous suprachoroidal hemorrhage, vitrectomy

## Abstract

**Purpose:** To report a rare case of spontaneous suprachoroidal hemorrhage (SSCH) in a high myopia patient with rhegmatogenous retinal detachment (RRD) and successful treatment.

**Methods:** We present a case of SSCH that occurred in a 73 woman with high myopia with RRD and discuss the results of a systemic review of the literature published from 1999 to 2017.

**Results:** Phacoemulsification without intraocular lens implantation and vitrectomy combined with silicone oil injection was performed and retinal detachment and choroidal detachment were reattached after oil removed. In the literature review, we found that among a total of 36 patients (37 eyes), acute secondary glaucoma was a complication in 70.3% (26 eyes) of the cases, and over half of the cases (24 eyes, 64.9%) were treated with surgery. Eighteen cases (50%) were characterized by systemic hypertension and 21 cases (58.3%) had abnormal hemostasis. Age-related macular degeneration (ARMD) was the most common (12 eyes, 32.4%) ocular disease and was followed by glaucoma (7 cases, 18.9%). Visual acuity was classified as hand motion (HM) or worse in 25 eyes (out of 34 eyes, 73.5%) at initial presentation and in 25 eyes (out of 36 eyes, 69.4%) upon final examination. Nine cases experienced significant visual improvement, including six that underwent vitrectomy.

**Conclusion:** Advanced age, systemic anticoagulation, and hypertension are strong risk factors. RRD associated with massive SSCH is an extremely rare event. Vitrectomy and choroidal blood drainage can effectively remove suprachoroidal hemorrhage (SCH) and promote retinal reattachment in these eyes. However, the final visual prognosis usually remains poor.

## Introduction

Suprachoroidal hemorrhage (SCH) is usually associated with ocular surgery or ocular trauma. Spontaneous suprachoroidal hemorrhage (SSCH) is much more unusual. In most reported spontaneous cases, the patients were predisposed to bleeding because of inherited blood dyscrasia, age-related macular degeneration (ARMD), or the use of systemic antithrombotic therapy (e.g., antiplatelet, anticoagulation, or thrombolytic agents) [1]. Here, we report a case of SSCH in a high myopia patient with rhegmatogenous retinal detachment (RRD) and choroidal detachment. We also review the relevant literature regarding predisposing factors, the clinical course, and visual outcomes with the aim of preventing the occurrence of SSCH and providing appropriate management strategies.

## Case report

**Figure 1:**
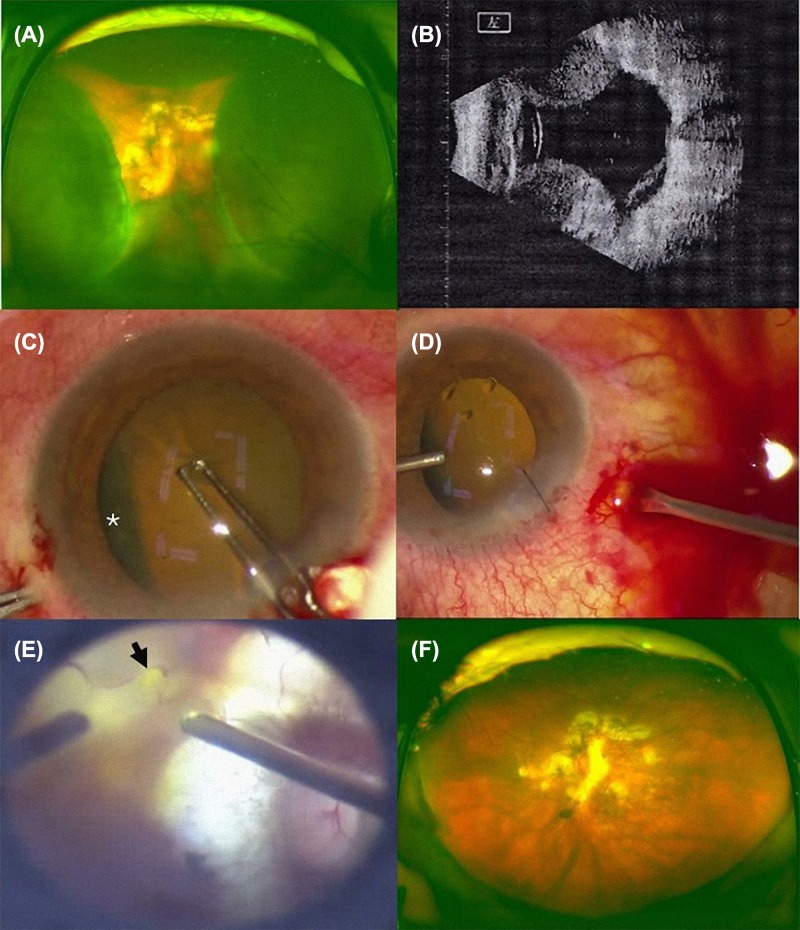
Clinical data of patient **(A**) Fundus examination revealed retinal detachment with choroidal detachment. (**B**) B-ultrasound revealed retinal detachment with choroidal detachment. (**C**) The choroidal detachment (white star) was clearly visible before phacoemulsification. (**D**) During the surgery, a copious, thick flux of blood flowed out of the 20G cannulas and was followed by a yellowish liquid. (**E**) A 1/5PD hole was found in the arch of the vascular arch below the macula (black arrow). (**F**) After surgical treatment, the retinal detachment and choroidal detachment were reduced.

A 73-year-old Chinese female presented with blurred vision in the left eye for 50 days without any obvious inducement. Her medical history included high myopia for more than 50 years, but she had never had diabetes, hypertension, or other systemic illness. On admission, her blood pressure was 100/60 mmHg, her visual acuity was light perception, and her intraocular pressure (IOP) was 5 mmHg in the left eye. An ocular examination performed on the left eye showed a flare and was positive for cells in the anterior chamber. A fundus examination and B-ultrasound revealed retinal detachment with choroidal detachment (Figure 1A, B).

All hematological and biochemical tests were within normal limits. After surgical contraindications were excluded, we performed a combined phacoemulsification without intraocular lens implantation and vitrectomy in combination with silicone oil injection. The choroidal detachment (Figure 1C, star) was clearly visible before phacoemulsification was performed. After phacoemulsification, 20G and 23G vitrectomy cannulas were placed 3.5 mm from the limbus. The 20G cannula was left open, and the infusion line was placed in the anterior chamber through a clear corneal paracentesis with a bottle height of 40 mmHg. As soon as the infusion line was opened, a copious, thick flux of blood flowed out of the 20G cannulas and was followed a yellowish liquid (Figure 1D). As the blood flow continued, the choroidal detachment visibly recessed. Though there was tight adhesion between the posterior vitreous and retina, the posterior vitreous cortex was completely separated from the inner surface of the retina. A 1/5PD hole was found in the arch of the vascular arch below the macula (Figure 1E, black arrow), and a full vitrectomy combined with intravitreal silicone oil tamponade was performed. After surgical treatment, the retinal detachment and choroidal detachment were reduced (Figure 1F). After treatment with a silicone oil tamponade, visual acuity was hand motion (HM) and counting fingers after 1 day and 1 month, respectively. After 3 months, the silicone oil was removed, and recurrent retinal detachment occurred because of proliferative vitreoretinopathy (PVR). A secondary surgery was performed to peel the preretinal membrane and apply silicon oil, and the silicone oil was removed 3 months later. Finally, the patient showed a good reattached choroidea and retina and achieved a visual acuity of 20/2000.

## Materials and methods

A total of 29 articles (including 36 patients) published from December 1999 to March 2017 were reviewed and analyzed according to clinical course, risk factors, management, and final outcomes. Studies in which SSCH happened during or immediately after the surgery were excluded.

Including our case [1–28] (Table 1), 13 men and 23 women (37 eyes after 2 cases with missing data were excluded) were analyzed. One case had bilateral involvement for 1 year. The ages of the patients ranged from 27 to 90 years old [mean ± standard deviation (SD) 67.9 ± 13.6 years old]. The IOP on initial presentation ranged from 3 to 70 mmHg in 32 recorded eyes (mean ± SD 39.8 ± 14.7 mmHg), and four patients did not have a high IOP on the first visit prior to SSCH progression. Among five eyes without IOP data, high IOP was listed as >50 mmHg in one case, and two had corneal perforations that were assumed to have resulted from glaucoma. In all, acute glaucoma was noted in 28 eyes (75.7%). Two cases of low IOP (3–5 mmHg) were high myopia with RRD.

**Table 1:** Review of previous reports of sapantaneous choroidal hemorrhage

	Reference (year)	Age (year)	Sex	Eye	MH	OH	Anticoagulant agent	Initial VA	Initial IOP (mmHg)	Management	Final VA
1.	Wong [2], 1999	76	M	R	HTN, IHD, DM	IOL	Heparin	CF	60	Medications	NLP
2.	Ophir [3], 2001	90	F	R	IHD, DM	Glaucoma	None	NLP	Perforation	Eviseration	–
3.	Chen [1], 2001	57	M	R	HTN, CBE, DM	AMD	None	HM	50	Medicaions	NLP
		78	M	L	CAD	AMD	None	NLP	50	Sclerotomy	NLP
		55	M	R	HTN	AMD	None	NLP	42	Sclerotomy	HM
		67	F	L	HTN, DM, VHC	AMD	None	NA	70	Sclerotomy	N/A
4.	Dandekar [4], 2001	62	F	L	RHD	None	Warfarin	NA	NA	Medications	LP
5.	Neudorfer [5], 2002	84	F	L	IHD,	IOL	Enoxaparin	CF	70	Medications	20/100
6.	Knox [6], 2002	81	F	R	A-Fib	AMD	Warfarin	LP	36	Iridotomy	NLP
7.	Goldsmith [7], 2003	74	F	L	HTN, CRF, AL	Glaucoma	None	NLP	Perforation	Evisceration	–
8.	Chak [8], 2003	78	F	L	HTN	High myopia	Aspirin	6/12	20	Medications	6/12
9.	Hammam [9], 2003	65	M	R	None	None	None	6/24	38	Medications	6/5
10.	Yang [10], 2003	84	M	R	HTN	AMD	None	HM	43	Sclerotomy	NLP
		84	F	R	HTN, A-Fib	AMD, IOL	Warfarin	HM	26	Sclerotomy	NLP
		66	F	L	MVR	AMD	Warfarin	LP	40	Sclerotomy	NLP
		85	M	L	HTN	AMD	None	HM	18	Sclerotomy	NLP
		79	F	L	HTN, A-Fib	AMD, IOL	Warfarin	LP	29	Sclerotomy	HM
11.	Maguluri [11], 2005	52	M	R	Hemophilia	None	None	HM	27	PPV, PPL, enucleation	–
12.	Barsam [12], 2005	86	F	R	DM, IHD	Glaucoma, IOL	Aspirin	NLP	>50	Medications	HM
13.	Saeed [13], 2007	27	F	L	DM, hemodialysis	DR, IOL	tPA	CF	48	PPV, SO	6/12
14.	Lee [14], 2007	81	F	L	HTN,	None	Aspirin, plavix	NLP	58	Enucleation	–
15.	Tajika [15], 2008	32	M	L	CRF	None	None	0.01	34	PPV	0.8
16.	Chandra [16], 2009	84	M	L	HTN, CBS, PE	AMD, Glaucoma	Warfarin	NLP	44	Medications	NLP
17.	Fukuchi [17], 2009	46	F	L	HTN	NA	NA	NA	55	Medications	NA
18.	Chen [18], 2009	86	M	R	IHD, DVT	Glaucoma, AMD	Aspirin	NLP	45	Sclerotomy, evisceration	–
19.	Lim [19], 2011	75	F	L	Aortic aneurysms	Hypermetropic	None	HM	70	Paracentesis, enucleation	–
20.	Nadarajah [20], 2012	71	F	NA	HLD, HTN	Glaucoma	NA	LP	NA	Sclerotomy	6/48
21.	Nguyen [21], 2012	24	F	L	Cystic fibrosis, DM	DR	Warfarin, heparin	HM	34	Medications	NLP
22.	Kim [22], 2013	53	F	R	PD, HTN, CRF, DM	Vitrectomized	None	CF	19	Sclerotomy	20/40
23.	Srikanth [23], 2013	57	F	R	DLD	NA	NA	NLP	44	Perforaion, evisceration	–
24.	Zhang [24], 2014	59	F	R	None	High myopia	None	LP	3	Phaco, PPV, SO	CF
		61	F	R	None	High myopia	None	HM	5	PPV, SO	20/400
25.	Andreatta [25], 2016	90	F	L	A-Fib	AMD	Warfarin	HM	55	Medications	LP
26.	Hsiao [26], 2016	64	M	R	HTN, BS,	None	Clopidogrel bisulfate	NLP	59	Iridotomy, sclerotomy	NLP
27.	Albert [27], 2017	62	F	R, L	HTN, DM, HLD	Glaucoma	None	20/25 (R), 20/200 (L)	17	Medications	20/40(R), 20/20(L)
28.	Atdignysx [28], 2017	68	F	L	HTN, AL, HF	None	Warfarin	LP	50	Sclerotomy	NLP

MH: medical history; OH: ocular history; VA: visual acuity; IOP: intraocular pressure; M: male; F: female; R: right; L; left; HTN, hypertension; IHD: ischemic heart disease; DM: diabetes mellitus; CAD: coronary arterial disease; CBE: cerebrovascular episode; VHC: viral hepatitis C; NA: not available; RHD: rheumatic heart disease; A-Fib: atrial fibrillation; MVR: mitral valve replacement; CRF: chronic renal failure; BS: brainstem stroke; AL: arteriosclerosis; AMD: age-related macular degeneration; tPA: tissue plasminogen activator; CBS: cardiac bypass surgery; PE: pulmonary embolus; IOL: intraocular lens; DR: diabetic retinopathy; HLD: hyperlipidemia; DVT: deep vein thrombosis; PD: peritoneal dialysis; DLD: decompensated liver disease; HF: heart failture; CF: count fingers; HM: hand motions; LP: light perception; NLP: no light perception; PPV: pars plana vitrectomy; PPL: pars plana lentectomy; SO: silicone oil.

Table 2 summarizes the systemic factors associated with the development of SSCH. Hypertension was the most frequent systemic disease (18 cases, 50%) and was followed by cardiovascular or cerebrovascular disease (17 cases, 47.2%) and diabetes mellitus (9 cases, 25%). The Valsalva maneuver was noted in four cases (10.8%) prior to the episode. Chronic renal failure (CRF, 3 cases), decompensated liver disease (1 case), hemodialysis (1 case), peritoneal dialysis (1 case) and hemophilia (1 case) might have contributed to abnormal hemostasis. Anticoagulant, antiplatelet, and thrombolytic agents were used in 17 cases (47.2%) and included warfarin (9 cases), aspirin (4 cases), heparin (3 cases), clopidogrel (1 case), plavix (1 case) and a tissue plasminogen activator (tPA, 1 case). A combination of two anticoagulants was noted in two cases (Table 1). Table 2 also shows the associated ocular diseases. The most common factor was ARMD (12 eyes, 32.4%), which was followed by glaucoma (7 eyes, 18.9%) and pseudophakia (6 eyes, 16.2%). High myopia (3 eyes, 8.1%) and diabetic retinopathy (1 eye, 2.7%) were seldom associated.

**Table 2:** Summary of previous reports of sapantaneous choroidal hemorrhage

Characteristics	Values
**Demographic data**	***n***
Sex (M/F)	13/23
Eye (R/L)	17/20
Average age (year ± SD)	67.9 ± 16.7
Age range (year)	27-90
**Medical history**	***n* (%)**
Hypertension	18 (50)
Diabetes mellitus	9 (25)
Ischemic heart disease	5 (13.9)
Atrial fibrillation	4 (11.1)
Chronic renal failure	3 (8.3)
Arteriosclerosis	2 (5.6)
Hyperlipidemia	2 (5.6)
Hemophilia	1 (2.8)
Hemodialysis	1 (2.8)
Aortic aneurysms	1 (2.8)
Cystic fibrosis	1 (2.8)
Cerebrovascular episode	1 (2.8)
Coronary arterial disease	1 (2.8)
Viral hepatitis C	1 (2.8)
Rheumatic heart disease	1 (2.8)
Mitral valve replacement	1 (2.8)
Deep vein thrombosis	1 (2.8)
Peritoneal dialysis	1 (2.8)
Decompensated liver disease	1 (2.8)
Brainstem stroke	1 (2.8)
None	3 (8.3)
**Anticoagulant agent**	***n* (%)**
Warfarin	9 (25)
Aspirin	4 (11.1)
Heparin	3 (8.3)
Tissue plasminogen activator	1 (2.8)
Clopidogrel bisulfate	1 (2.8)
Plavix	1 (2.8)
None	16 (44.4)
Not available	3 (8.3)
**Ocular history**	***n***** (%)**
Age-related macular degeneration	12 (32.4)
Glaucoma	7 (18.9)
Pseudophakia	6 (16.2)
High myopia	3 (8.1)
Hypermetropic	1 (2.7)
Vitrectomized	1 (2.7)
Diabetic retinopathy	1 (2.7)
None	9 (24.3)
Not available	2 (5.4)
**Treatment**	***n* (%)**
Conservative treatment	13 (35.1)
Sclerotomy	11 (29.7)
Eviseration/enucleation	7 (18.9)
Vitrectomy	4 (10.8)
Laser iridotomy	2 (5.4)
Paracentesis	1 (2.7)

M: male;F: female; R: right; L: left; SD: standard deviation.

In addition to m edication, 24 eyes (64.9%) received surgical intervention (Table 2). Blood drainage with sclerotomy or vitrectomy was performed in 15 eyes (40.5%) from the 2nd day to over 30 days after the episode. Laser iridotomy performed in 2 cases (5.4%), while paracentesis was performed in one case (2.7%) to reduce IOP. Two cases (5.4%) had subsequent global perforations, and one of these was treated with evisceration. The other five eyes underwent evisceration or enucleation due to corneal perforation or pain or recurrent SSCH following drainage.

At the initial examination, visual acuity was HM or worse in 25 of the 34 recorded eyes (73.5%), including 10 eyes (28.6%) that presented with no light perception. Finally, the final visual acuity was HM or worse in 25 eyes (of 36 eyes, 69.4%), and no light perception was found in 20 eyes (55.6%), including six phthitic eyes and seven anophthalmic cases. Nine cases experienced significant visual improvement, and six of these improved after vitrectomy. Fair visual acuity on presentation was noted in only two cases (20/25, 6/12 and 20/40, 6/12 following medical therapy).

## Discussion

SCH is a vision-threatening complication associated with ocular trauma or certain surgical procedures, such as cataract extraction, glaucoma filtering surgery, penetrating keratoplasty, and vitreoretinal surgery [29–33]. However, SSCH is uncommon and has previously been described in only isolated case reports. The recognized risk factors for SSCH include systemic diseases treated with anticoagulants [6,7] and ARMD treated with anticoagulants [1,2,10]. RRD associated with SSCH is rare.

The exact cause for the development of SSCH in RRD patients is unclear. Speaker et al. [34] were the first to publish a report (in 1991) showing that increased axial length is a significant independent risk factor for SCH in intraocular surgery because a longer axial length causes choroidal vascular fragility to increase. Hypertension, arteriosclerosis, and advanced age have been widely reported to be systemic risk factors for SCH in a surgical setting, but the association between these risk factors and spontaneous hemorrhage is unknown [35,36]. In 2003, Chak and Williamson [8] reported a case of SSCH with high myopia and aspirin and suggested that high myopia and using anticoagulants may be risk factors for SSCH. Zhang et al. [24] reported six cases of RRD and massive SSCH in eyes that were all associated with high myopia and increased ocular length. In our study, this patient had no history of systemic disease but was associated with an increased axial length of more than 30 mm. Therefore, the risk factor that is suspected to have predisposed this patient to massive SSCH in RRD may be a long axial length. An increased axial length may also be related to increased choroidal vascular fragility. Severe myopia was one of the main risk factors for RRD associated with choroidal detachment. Moreover, a dramatic drop in IOP increase the risk of developing SSCH in patients with RRD associated with choroidal detachment myopia. Usually, massive SCH leads to a sharp increase in IOP due to the increase in ocular contents [1,3,5,12,18]. In the present study, choroidal detachment was caused by retinal detachment in patients with high myopia, and SCH was caused by low IOP and fragile vascular elasticity, so that there was no increase in IOP in this patient.

Surgical drainage of SCH without RRD has been reported in a few isolated papers, but the clinical effects and optimal timing of this surgical intervention remains uncertain [10,20,37,38]. Yang proposed that surgical intervention may have value for relieving pain and elevated IOP but has not been shown to be beneficial for visual outcomes. In our review, at the initial examination, the patients’ visual acuity was HM or worse in 25 of the 34 recorded eyes (73.5%), including 10 eyes (28.6%) that presenting with no light perception. In the end, the final visual acuity was HM or worse in 25 eyes (of 36 eyes, 69.4%), and no light perception was found in 20 eyes (55.6%), including 6 phthitic eyes and seven anophthalmic cases. Nine cases experienced significant visual improvement, and six of these improved after vitrectomy. Fair visual acuity on presentation was noted in only two cases (20/25, 6/12 and 20/40, 6/12 following medical therapy). Our patient was associated with RRD and high myopia. These basic lesions can themselves also cause vision loss. We took advantage of 20G and 23G vitrectomy cannulas to ensure sclerotomies of known and reliable diameter and consistent patency throughout all surgical maneuvers. During infusion and vitrectomy, the sclerotomies remained functional and permitted continuous blood flow out of the suprachoroidal space. This method has also been described in other studies that used 23G or 25G cannulas [39]. The use of 20G and 23G cannulas also allows the very quick, safe, and easy closure of the sclerotomy when needed. After vitrectomy, suprachoroidal blood drainage, photocoagulation, phacoemulsification and silicon oil tamponade, and retina reattachment are performed. PVR causes retinal detachment after oil extraction. In a secondary surgery preretinal membrane peeling and silicon oil retention were performed. Finally, this patient showed a good reattached choroidea and retina and achieved a visual acuity of 20/2000 after the silicone oil was removed. We hypothesize that the PVR was caused by two factors. First, tight adhesion between the posterior vitreous membrane and retina makes the posterior vitreous cortex difficult to clear. Second, massive SCH can also stimulate PVR development.

There is some disagreement regarding the timing of surgery. Some authors suggest waiting 10–14 days for the clot to liquefy, whereas others advocate early surgical intervention to achieve better anatomical and visual outcomes [40]. Waiting for spontaneous resolution can result in retinal detachment when there is vitreous incarceration, which can in these cases lead to a poor visual prognosis. In cases of extensive hemorrhage, patients have sustained vision loss from chronic atrophy or phthisis bulbi in the absence of prompt surgical intervention. Prompt drainage may provide the best chance for maintaining useful vision. In some studies, the mean time interval was 11 days (range, 6–20 days) [40,41]. Generally, a longer duration of appositional SCH has been shown to result in poorer visual outcomes [42]. In accordance with previous studies, we also suggest that the time interval should not exceed 14 days.

The literature review presented in this work indicates that SSCH is highly associated with hypertension, systemic anticoagulation, and ARMD. If medication cannot be withheld, general practitioners or cardiologists should consult an ophthalmologist for a complete ophthalmic examination prior to treatment, and they should also inform their patients of the possible risk factors associated with SSCH. In the case of patients with intractable pain, surgery should be performed. The limitations of our review include its retrospective nature, the lack of a control group, and the limited number of cases. The definite relative risk of each factor therefore could not be clearly determined.

## Conclusions

Overall, RRD associated with massive SSCH is an extremely rare event. The most common risk factor is long axial length. Vitrectomy and choroidal blood drainage can effectively remove SCH and promote retinal reattachment in these eyes. However, the final visual acuities are generally poor.

## Availability of data and material

All data supporting our findings will be shared upon request, although the majority is contained within the manuscript.

## Supporting information

**Supplementary Video:** 

## Abbreviations

ARMD: age-related macular degeneration
HM: hand motion
IOP: intraocular pressure
RRD: rhegmatogenous retinal detachment
SCH: suprachoroidal hemorrhage
SSCH: spontaneous suprachoroidal hemorrhage
